# VirusViz: comparative analysis and effective visualization of viral nucleotide and amino acid variants

**DOI:** 10.1093/nar/gkab478

**Published:** 2021-06-09

**Authors:** Anna Bernasconi, Andrea Gulino, Tommaso Alfonsi, Arif Canakoglu, Pietro Pinoli, Anna Sandionigi, Stefano Ceri

**Affiliations:** Dipartimento di Elettronica, Informazione e Bioingegneria, Politecnico di Milano, Via Ponzio 34/5, 20133 Milano, Italy; Dipartimento di Elettronica, Informazione e Bioingegneria, Politecnico di Milano, Via Ponzio 34/5, 20133 Milano, Italy; Dipartimento di Elettronica, Informazione e Bioingegneria, Politecnico di Milano, Via Ponzio 34/5, 20133 Milano, Italy; Dipartimento di Elettronica, Informazione e Bioingegneria, Politecnico di Milano, Via Ponzio 34/5, 20133 Milano, Italy; Dipartimento di Elettronica, Informazione e Bioingegneria, Politecnico di Milano, Via Ponzio 34/5, 20133 Milano, Italy; Quantia Consulting S.r.l., Via Petrarca 20, 22066, Mariano Comense, Como, Italy; Dipartimento di Elettronica, Informazione e Bioingegneria, Politecnico di Milano, Via Ponzio 34/5, 20133 Milano, Italy

## Abstract

Variant visualization plays an important role in supporting the viral evolution analysis, extremely valuable during the COVID-19 pandemic. VirusViz is a web-based application for comparing variants of selected viral populations and their sub-populations; it is primarily focused on SARS-CoV-2 variants, although the tool also supports other viral species (SARS-CoV, MERS-CoV, Dengue, Ebola). As input, VirusViz imports results of queries extracting variants and metadata from the large database ViruSurf, which integrates information about most SARS-CoV-2 sequences publicly deposited worldwide. Moreover, VirusViz accepts sequences of new viral populations as multi-FASTA files plus corresponding metadata in CSV format; a bioinformatic pipeline builds a suitable input for VirusViz by extracting the nucleotide and amino acid variants. Pages of VirusViz provide metadata summarization, variant descriptions, and variant visualization with rich options for zooming, highlighting variants or regions of interest, and switching from nucleotides to amino acids; sequences can be grouped, groups can be comparatively analyzed. For SARS-CoV-2, we manually collect mutations with known or predicted levels of severity/virulence, as indicated in linked research articles; such critical mutations are reported when observed in sequences. The system includes light-weight project management for downloading, resuming, and merging data analysis sessions. VirusViz is freely available at http://gmql.eu/virusviz/.

## Graphical Abstract

**Graphical Abstract:**
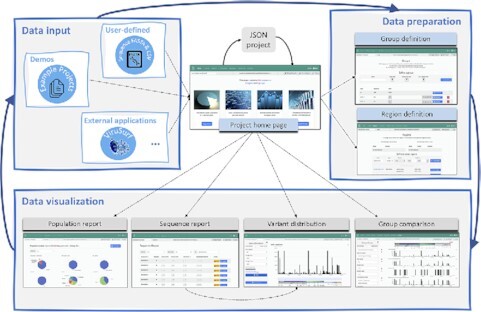
VirusViz accepts as input user-defined viral sequences or results from external applications; it supports data preparation by defining groups and regions of interest for producing powerful data visualization and comparisons of variants.

## INTRODUCTION

With the progress of the COVID-19 pandemic, the SARS-CoV-2 virus is undergoing adaptive evolution that improves its fitness to the host’s environment—the human population (1). Unprecedented interest follows the sequencing of new variants, as scientists want to understand better how fast they spread, if they enhance the virus transmission, if they correlate with heavier or milder forms of the disease, and if they could hamper the effectiveness of currently authorized and future vaccines.

Even if the term ‘variant’ has been used to denote particular strains or lineages, in the following we use it with the traditional meaning of single nucleotide variant or amino acid change with respect to the the reference sequence. Taking advantage of our experience in data integration for human genomics (2,3), we designed the Viral Conceptual Model (4) and used its structure to develop ViruSurf (5) (http://gmql.eu/virusurf/)—a database hosting viral sequences collected from GenBank (6), COG-UK (7) and NMDC (http://nmdc.cn/). This database hosts the largest freely queryable collection of SARS-CoV-2 variants deposited worldwide (as of 11 May 2021, about 714K sequences). The data is frequently updated by an incremental pipeline. ViruSurf allows to inspect and download query results, but does not further support users in data visualization, exploration, and analysis.

VirusViz, hereby presented, complements our database and search system; it is a web-based application to visualize and inspect viral variants (currently supporting SARS-CoV, MERS-CoV, Dengue, and Ebola) over populations and their sub-populations, which can be directly invoked from within the ViruSurf result page, thereby enabling a seamless analysis and visualization of query results.

In addition to database results, VirusViz can be used to analyze given viral populations that are not deposited to a public database; for satisfying this need, we offer the option of directly inputting new viral sequences in FASTA format, with metadata provided within a single CSV file. A bioinformatic pipeline extracts their nucleotide and amino acid variants as well as the impact of each nucleotide variant and the lineage of SARS-CoV-2 sequences. When the pipeline terminates, the new sequences and their variants can be inspected.

Several user-provided inputs and database query results can be explored over time. For saving data analysis projects, we designed a persistent structure in standard JSON format that encodes results from database queries or from the bioinformatic pipeline; the JSON file is progressively enriched during the user’s analysis, e.g. by keeping track of the defined sub-populations or highlighted regions. Thanks to this encoding, data analysis sessions can be suspended, resumed or merged at any time. Such functionality may be exploited for merging results collected at different time points, as well as from private data and public data.

While any variant of a given population can be observed within VirusViz, certain variants – that could have biological relevance – continually emerge from the current literature. Specifically for SARS-CoV-2, we systematically collect and classify these variants within a manually curated *knowledge base*, providing external links to related articles; they are reported when observed in specific sequences.

The user may also highlight particular regions describing relevant portions of the genome (e.g. the Receptor Binding Domain in the Spike protein) or sets of variants characterizing notable strains (e.g. those denoted as the *UK, Brazilian* and *South African variants*, which are getting the press attention at the time of writing).

### Related Work

Many search systems for viral sequences and their variants were built since the outbreak of the COVID-19 pandemic (8). Some of these are equipped with engines for processing user-input sequences and annotating them with variants and other features (e.g. CoV-GLUE (9) and 2019nCoVR (10)). A number of resources have focused on interactive visualization regarding SARS-CoV-2, including CoVMT (11), GESS (12), coronApp (13), Pango lineages, CoVariants, hcov19-variants (embedded within the GISAID website), cov.lanl, outbreak.info, and CoV Genetics.

Most of the mentioned resources have a focus only on specific accruing mutations. Table 1 represents a comparison of such resources, considering provided features along four main categories: accepted inputs, annotation services applied on the data, criteria for population design, visualization analysis. To the best of our knowledge, VirusViz is the first completely open access web application supporting variant analysis and visualization for several viral species (not only SARS-CoV-2) for all types of variants (not only amino acid ones), accepting input from both public sequences (selected by users through ViruSurf) and user-provided sequences (processed through a bioinformatic pipeline), integrated with a curated knowledge base of variants of interest (continuously updated). In particular, VirusViz stands out for allowing a comparative inspection of variant distributions.

**Table 1.** Comparison of resources for searching and visualizing viral sequences. $\checkmark$indicates that the resource presents the full functionality; else, we briefly indicate in which way the functionality is limited. For example – for CoVariants – aa/VoI in ‘amino acid analysis’ means that it is available only for particular amino acid changes and Variants of Interest (or notable variants); country in ‘define populations by metadata’ means that it may be performed using only country as a filter

		CoV-GLUE	2019nCoVR	CoVMT	GESS	coronApp	Pango lineages^a^	CoVariants^b^	hcov19 variants^c^	cov.lanl^d^	outbreak info^e^	CoV Genetics^f^	**VirusViz**
	Features	(9)	(10)	(11)	(12)	(13)							-
input	process user-input FASTA	$\checkmark$	$\checkmark$	-	-	$\checkmark$	-	-	-	$\checkmark$	-	-	$\checkmark$
	process user-input metadata	-	-	-	-	-	-	-	-	-	-	-	$\checkmark$
	support other viruses	-	-	-	-	-	-	-	-	-	-	-	$\checkmark$
annotation	driven by nuc/aa/variant	aa	aa	aa	nuc	nuc+aa	var	var	var	aa	aa+var	aa	nuc+aa
	nucleotide analysis	$\checkmark$	$\checkmark$	$\checkmark$	$\checkmark$	$\checkmark$	-	-	-	$\checkmark$	-	-	$\checkmark$
	amino acid analysis	$\checkmark$	$\checkmark$	$\checkmark$	$\checkmark$	$\checkmark$	VoI	aa/VoI	VoI	$\checkmark$	$\checkmark$	$\checkmark$	$\checkmark$
	co-occurrence analysis	-	-	-	$\checkmark$	-	-	aa/VoI		$\checkmark$	$\checkmark$	$\checkmark$	$\checkmark$
	integrate ext. knowledge	-	-	-	-	-	-	$\checkmark$	-	-	$\checkmark$	-	$\checkmark$
design	define populations by metadata	-	country	-	-	-	-	country	-	-	country	country	$\checkmark$
	define populations by mut/var	-	nuc	-	-	-	VoI	aa/VoI	-	-	aa/VoI	Spike aa	$\checkmark$
analysis	mutations on genome	-	$\checkmark$	$\checkmark$	$\checkmark$	top 10	-	-	-	$\checkmark$	-	$\checkmark$	$\checkmark$
	mut/var through time	-	$\checkmark$	$\checkmark$	-	-	-	$\checkmark$	$\checkmark$	-	$\checkmark$	$\checkmark$	-
	comparison of distributions	-	-	-	-	-	-	$\checkmark$	-	-	-	-	$\checkmark$

Some resources do not have a related publication, their links are: ^a^https://cov-lineages.org/; ^b^http://covariants.org/; ^c^https://www.gisaid.org/hcov19-variants/; ^d^https://cov.lanl.gov/; ^e^https://outbreak.info/; ^f^https://covidcg.org/.

## MATERIALS AND METHODS

### VirusViz pipeline for processing user-provided FASTA sequences

The annotation and variant calling used for user-provided sequences is inherited from ViruSurf (5). To assign the appropriate reference alignment, the virus species must be indicated. Each sequence is aligned to the reference genome using the Needleman–Wunsch algorithm with affine gap penalty, and nucleotide variants with associated putative impact are computed using SnpEff (14); coding regions are translated to amino acid sequences and variants are inferred.

More advanced analysis is implemented in VirusViz: we added the assignment of each SARS-CoV-2 sequence to its lineage using Pangolin (15) and we defined the notion of *distance between two sequences* as the counter of the variants that they do not have in common. Distance is used to identify the closest sequences to a given one, and to compute the heterogeneity score–both for nucleotide and amino acid variants–as the average number of different variants between every pair of sequences in a population.

### Project management

The VirusViz web application is stateless; in order to support users in suspending, resuming, and merging data analysis sessions, we designed a light-weight project management. We represent the project’s state using a formatted JSON file; the full structure is shown in Supplementary Figures S1 and S2 and an example instance in Supplementary Figure S3. A JSON file with this format is used for uploading to VirusViz either the result of a ViruSurf query or the output of the VirusViz pipeline.

While a data analysis session takes place, the project state is progressively aligned, thereby supporting a download to the user’s local file system that reflects the session evolution; JSON files can be re-uploaded at a later time. Thanks to this unified format, merge operations between projects can occur by combining two project states into one.

The system treats any virus input seamlessly. The taxonomy identifier of the viral species drives the selection of the reference genome, the regions of interest, and the virus image used below plots.

### Architecture

VirusViz is a rich-client web application. The back-end is used for processing user-provided FASTA sequences and extracting variants; it is developed in Python using the Flask micro-framework and exposes a REST API to the web interface.

The front end at the client side provides most of VirusViz functionalities; it is implemented using the JavaScript framework Angular.js and the library Apache ECharts v5 for advanced visualizations. The state of the computation is encapsulated in a JSON structure, it includes sequences, variants, groups, regions and all the other entities present in the interface. The client-side logic for filtering sequences and their variants, computing heterogeneity scores, managing the interactions with the user, and communicating with the back-end is developed in JavaScript and relies on several third-party open source tools and libraries, listed in https://github.com/DEIB-GECO/VirusViz/wiki/External-software. VirusViz is freely available without login requirements.

### SARS-CoV-2 knowledge base

Several published papers or preprint submissions claim particular effects of SARS-CoV-2 amino acid variants. We manually collected these resources through a systematic literature search and organized their content using carefully planned orthogonal categories, i.e. lower or higher protein stability, viral transmission, infectivity, disease severity, fatality rate, sensitivity to convalescent sera or monoclonal antibodies, binding affinity to host receptor. Variants are annotated with their effects and linked to the specific resources that define them. A complete discussion of the knowledge base design is outside the scope of this paper (a work in progress paper is available (16)). We just note that, in many cases, the reported effects are contrasting with each other and/or different in their significance, since the linked references may be peer-reviewed publications but also preprints.

## RESULTS

**Figure 1.**
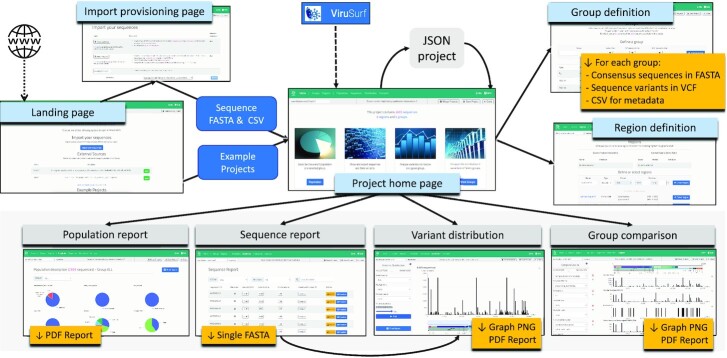
Description of the flow connecting various pages offered to users for controlling the interaction and producing reports. For each page, we indicate with yellow tags the different downloadable objects available for saving the obtained results. An example *Population report* is shown in Supplementary Figures S4 and S5.

The main interactions supported by VirusViz are highlighted in Figure 1. A *Landing* page (http://gmql.eu/virusviz/) includes several descriptions of example projects (documented in our WIKI, https://github.com/DEIB-GECO/VirusViz/wiki); their selection triggers the upload of the relevant files to VirusViz, leading the user to the *Project home* page.

From the *Landing* page users can also invoke external resources that produce data uploads for VirusViz: currently we support ViruSurf, but other databases are under development. The tool allows querying a rich collection of sequences based on their metadata. More advanced features allow to further filter results based on the presence of specific variants, both at the nucleotide and at the amino acid level. The produced result, consisting of a set of sequences—order of thousands—with their metadata and variants, is then used as input to VirusViz.

From the *Landing* page, users can also reach an *Input provisioning* page, where sequences can be uploaded. Input is provided as two cross-linked files: (i) a multi-FASTA file containing several sequences, each prefixed with a user-provided identifier and (ii) a CSV file for metadata, which includes user-provided identifiers within the first column and arbitrary metadata describing the sequence in the following columns. On completion of the pipeline, the user is taken to the *Project home* page; as the execution requires time, an optional email message can be set to notify completion.

The *Project home* page is the center of the data analysis steps; from here, it is possible to save/resume projects and merge an existing project to the current one. The page is linked through a top navigation bar to six pages:

- *Group definition*, for creating named sub-populations of interest;
- *Region definition*, for creating named regions of interest as (possibly discontinuous) intervals on the nucleotide sequence or on the amino acid sequence of a given protein;
- *Population report*, describing for a given group the metadata about sequences as well as aggregate information about their variants;
- *Sequence report*, describing the properties of each individual sequence;
- *Variant distribution*, for showing bar plots of nucleotide and amino acid variants;
- *Group comparison*, for comparatively showing bar plots of selected groups.

To illustrate VirusViz pages, we employ a *running example* (that can be opened directly from the VirusViz *Landing* page); all figures in this section describe specific aspects of the running example. The population has been obtained from ViruSurf by performing a query, executed on 9 February 2021, extracting 2695 SARS-CoV-2 sequences, with associated variants, collected in six US states between 1 November 2020 and 15 January 2021.

### Group definition

**Figure 2.**
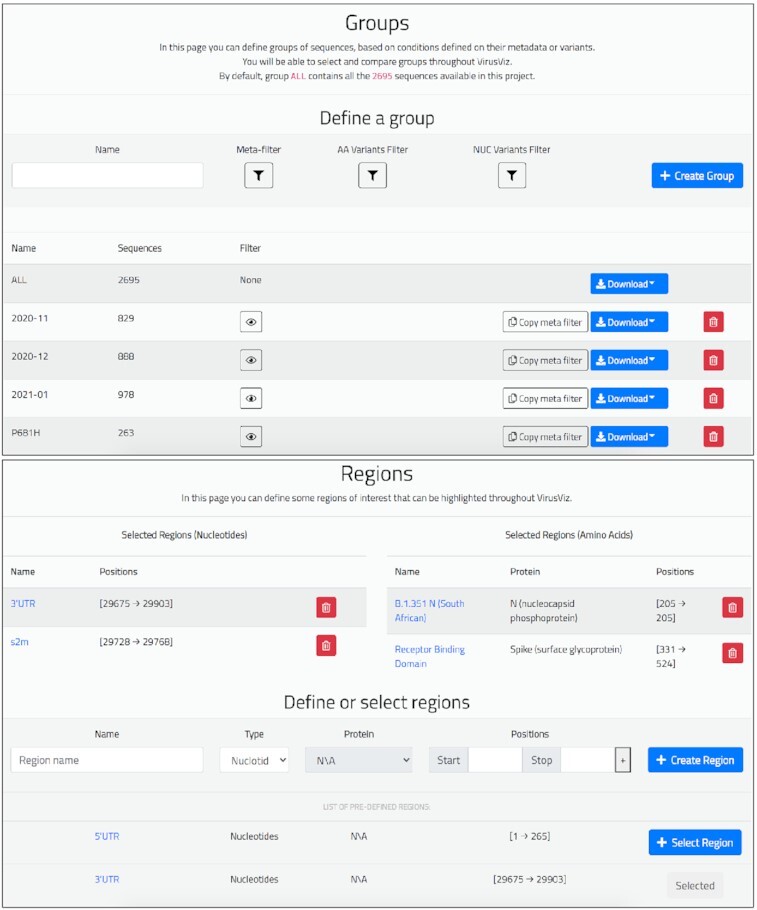
Top: *Group definition* page of the running example. We created three groups of similar sizes (829, 888 and 978) by selecting ranges of collection dates and one group containing sequences with the Spike change P681H (size 263). Bottom: Page for defining regions of interest. For nucleotides, we have added: (i) 3’ untranslated region (UTR) and (ii) Coronavirus 3’ stem-loop II-like motif (s2m); for amino acids: (iii) the Receptor Binding Domain and (iv) B.1.351 lineage variants in the N protein.

The *Group definition* page (see top part of Figure 2) is used for defining sub-populations by means of three kinds of filters, respectively on: (i) metadata attributes (selecting for a given ‘Attribute’ a subset of its ‘Values’), (ii) nucleotide variants (selecting the position and change type), and (iii) amino acid variants (selecting a protein and then the position and change type). Groups must be named, tentatively reflecting their selection criteria; the default group ‘ALL’ includes all the sequences. A table summarizes the properties of groups, including the number of sequences within the group and the filtering conditions used in their definition, which can be copied for creating a new group. The full group content can be downloaded in suitable formats.

### Region definition

The *Region definition* page (see bottom part of Figure 2) is used for collecting the definition of named regions of interest, to be highlighted within VirusViz plots; some of them can be read from a predefined list, others can be added by users by specifying either a set of ranges of nucleotide positions, or a set of ranges of amino acid positions within a specific protein. Added regions are displayed within two tables; these are used in the *Variant distribution* and *Group comparison* pages.

### Population report

**Figure 3.**
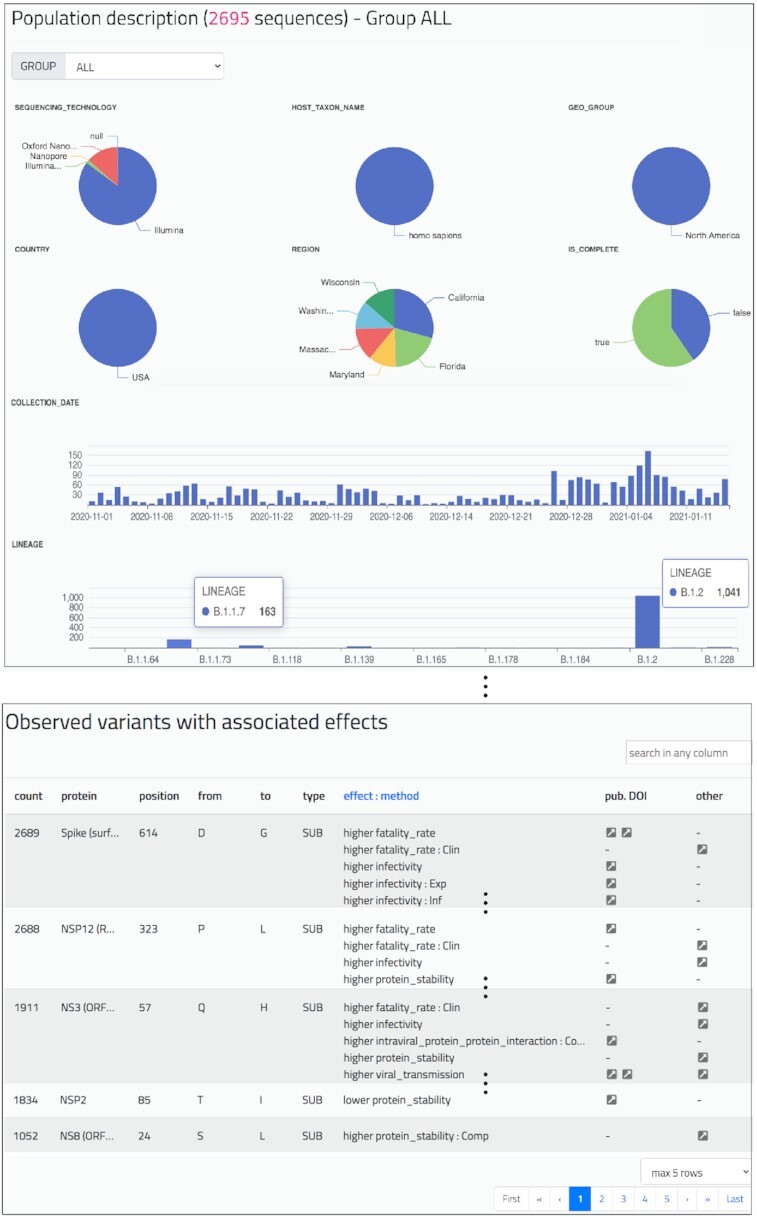
*Population report* of the group ‘ALL’ of the running example, with 2695 sequences. Pie charts show four sequencing technology values, six different US states, and the percentage of complete sequences. Bar plots describe amounts of sequences distributed by collection date (amounts increase during January 2021) and lineages (B.1.2 is prevalent; B.1.1.7 is present). For brevity we here omit the heterogeneity scores, and two tables of nucleotide and amino acid variants. The shown table highlights the presence of variants with linked effects, e.g., the two well-known changes D614G (Spike) and P323L (NSP12), the change Q57H (NS3)—for which multiple effects have been suggested – the change T85I in the NSP2, leading to lower protein stability, and the change S24L in the NS8, leading to higher protein stability (computationally predicted).

The *Population report* (see Figure 3) provides a general overview of a given sequence group through the following elements:

- Group selector (either the default ‘ALL’ group or a user-defined group): via a drop-down menu, the user can choose one group of interest and the whole content of the page is correspondingly updated.
- Pie charts for categorical metadata (with values and corresponding percentage within the selected group) and bar plots for numerical or dates metadata.
- Heterogeneity score indication, considering both nucleotides and amino acids; scores reflect the distance among variants of the sequences of the group, small values indicate that sequences have similar variants.
- Two tables with observed nucleotide and amino acid variants in the group, ordered by descending order of count.
- One table with interesting amino acid variants present in the group; these are retrieved from the knowledge base, including their effects and pointers to the descriptive literature resources.

### Sequence report

**Figure 4.**
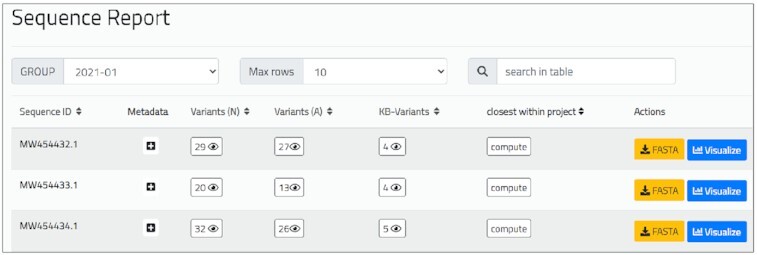
Excerpt of *Sequence report* of the ‘2021-01’ group of the running example. For the first sequence, i.e. MW454432.1, we list metadata (to be expanded by clicking on the ‘+’ symbol), 29 nucleotide variants, 27 amino acid variants, 4 notable variants. Users can request an ordered list of closest sequences; each sequence can be downloaded in FASTA format or opened and highlighted within the *Variant distribution* page.

The *Sequence report* (shown in Figure 4) is organized as a table. Each sequence corresponds to a row, for which we provide a pointer to the associated metadata (including the computed lineage) and counters linked to the lists of nucleotide variants (with corresponding impact annotations), amino acid variants and knowledge base variants. Another link can be clicked to compute a sequence’s closest sequences within the group, based on a distance metrics that counts pairs of different variants. For each sequence, the user may download the corresponding FASTA file (when available) or directly open the *Variant distribution* page, where variants present in the given sequence are highlighted to distinguish them from the other variants present in the group.

### Variant distribution

**Figure 5.**
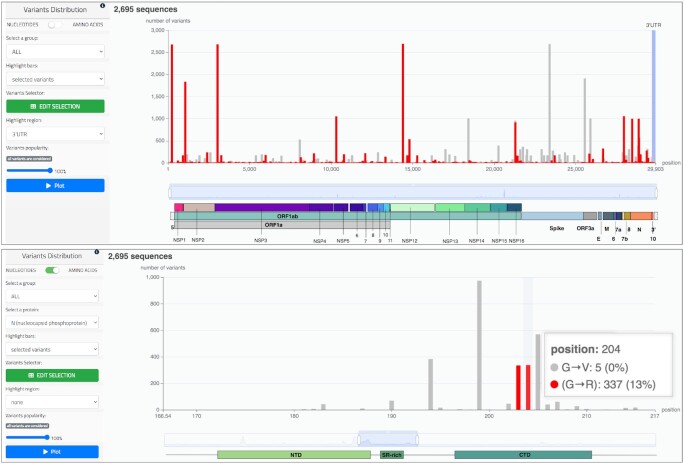
Top: bar plot representing the distribution of nucleotide variants of the running example. Bars highlighted in red indicate the number of sequences having a C to T mutation. The 3’ UTR of SARS-CoV-2 (bases 29675–29903) is highlighted with a blue background. Bottom: bar plot representing the distribution of amino acid variants in the nucleocapsid (N) protein of the running example. Using the slider at the bottom of the plot, we zoom to a particular range (166–217) of the protein where two variants from the knowledge base are highlighted in red (substitutions at positions 203 and 204); they are known for potentially lowering protein stability and improving viral transmission (21). By hovering on the bar at position 204, we observe a G to R change in 337 sequences, and a G to V change in five sequences.

This page provides the bar plot of the variants of a given group. The horizontal axis represents positions in the nucleotide or amino acid sequences; each bar’s height represents the number of sequences with a mutated nucleotide/amino acid in that position. Two working modes are available, respectively for nucleotides (top part of Figure 5) and for amino acids (bottom part of Figure 5). In the latter case, the user should also select a specific protein. After selection, a click on the ‘Plot’ button produces the graph.

The specific structure of the virus sequence is shown with a colored image at the bottom of the bar plot, adapted to the specific choice of virus species. A slider can be used for zooming to a preferred coordinate range. By hovering on the plot, the user may visualize the specific position represented by each bar and the precise sequence changes with its corresponding counts.

The user can select different elements to be highlighted in red in the plot: (i) variants belonging to a specific sequence (possibly chosen from the *Sequence report* table); (ii) specific variants (e.g. all C to T nucleotide substitutions, as shown in the top part of Figure 5); (iii) notable variants (e.g. the 203/204 substitutions in the N protein, as shown in the bottom part of Figure 5). The plot can be adjusted by excluding the most popular variants; this adjustment is advised to enhance bar visibility of less frequent variants when mixed with frequent but not relevant variants. One region, selected by means of a dedicated filter, can be added to the plot (e.g. the 3’ UTR region in the top part of Figure 5).

### Group comparison

**Figure 6.**
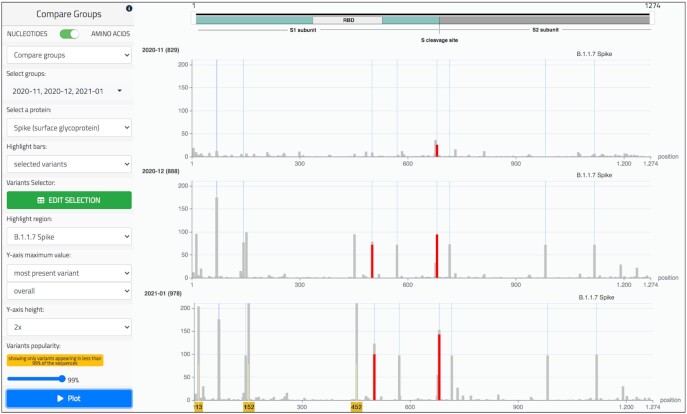
*Group comparison* page of the running example. The tracks represent sequences of the three groups filtered by date (November 2020, December 2020, and January 2021), as illustrated in Figure 2. The amino acid mode is selected and the Spike protein is observed. In the ‘Highlight region’ menu we select the variants that belong to the B.1.1.7 lineage (or ‘UK strain’), as defined in https://virological.org/t/preliminary-genomic-characterisation-of-an-emergent-sars-cov-2-lineage-in-the-uk-defined-by-a-novel-set-of-spike-mutations/563: HV 69-70 deletion, Y144 deletion, N501Y, A570D, P681H, T716I, S982A, D1118H; we note that all these bars become higher with the progression of time. Variants that are present in more than 99% of sequences are filtered to remove the confusing effect of frequent variants, such as D614G, now present in the great majority of sequences; then, the Y-axis maximum value is set to the height of the most present variant over all the tracks (i.e. 211 units in the third track). We further highlight in red two variants of particular concern: (i) the Asparagine (N) mutating into Tyrosine (Y) at position 501, possibly decreasing the neutralizing activity of convalescent plasma (22); (ii) the Proline (P) mutating into Histidine (H) at position 681, directly adjacent to the S1/S2 furin cleavage site, shown to promote the viral entry into lung cells (23). Note that the red portions only cover part of the bars, as the rest of them represent changes into different amino acids. In addition to the B.1.1.7 variants, we can observe additional variants whose presence is increasing: see positions 13, 152, 452 (marked with yellow number tags at the bottom). These amino acid changes identify the so-called ‘Californian variant’, which was still under definition in January 2021, the last period of observation (24). The figure is built by choosing the option ‘most present variant/overall’ and by expanding the Y-axis by a 2× coefficient.

As shown in Figure 6, this page displays a comparison of the variant frequency in multiple sub-populations, by presenting the corresponding bar plots in a stack. Similarly to the *Variant distribution* page, it provides two working modes, for nucleotides or amino acids. In addition, two options are available for building tracks, based either on the distinct values of a specific metadata attribute, or on user-defined groups.

Figure 6 shows an example for amino acids focused on the Spike protein, comparing tracks representing different user-defined groups. Many bars are observed in correspondence with the variants of the B.1.1.7 lineage (17), collectively named *UK variant* and highlighted with thin blue regions.

Several controls allow changing the plots in order to improve their comparative visualization; plots can be deleted, their order can be changed, their size can be normalized and scaled. In addition to filtering variants, as in the previous page, it is also possible to ease the visualization and comparative observations by changing the maximum value represented in the Y-axis of the tracks. The default setting is the number of sequences present in that group; the user may instead set the most present variant count as such maximum value on the track. Both options may be calculated over each single track or globally. Setting the Y-axis maximum value to the globally most present variant allows to compare bars based on their height (representing an absolute value), and it is therefore advised when tracks have similar sample sizes. Visual comparisons may be suggestive of hypotheses that need confirmation by means of accurate data analysis, carefully checking and eliminating any source of bias.

### Use case

#### South African variants in most recent US sequences

Finally, we simulate a scenario in which a user provides novel sequences to be analyzed within VirusViz. We selected from NCBI Virus portal (18) 36 sequences submitted on 16–17 February 2021 from four US States. We use the VirusViz pipeline to process the sequences formatted in a multi-FASTA file, along with their metadata in a CSV file; their processing may take from 30 minutes up to one hour depending on the current load of our servers.

**Figure 7.**
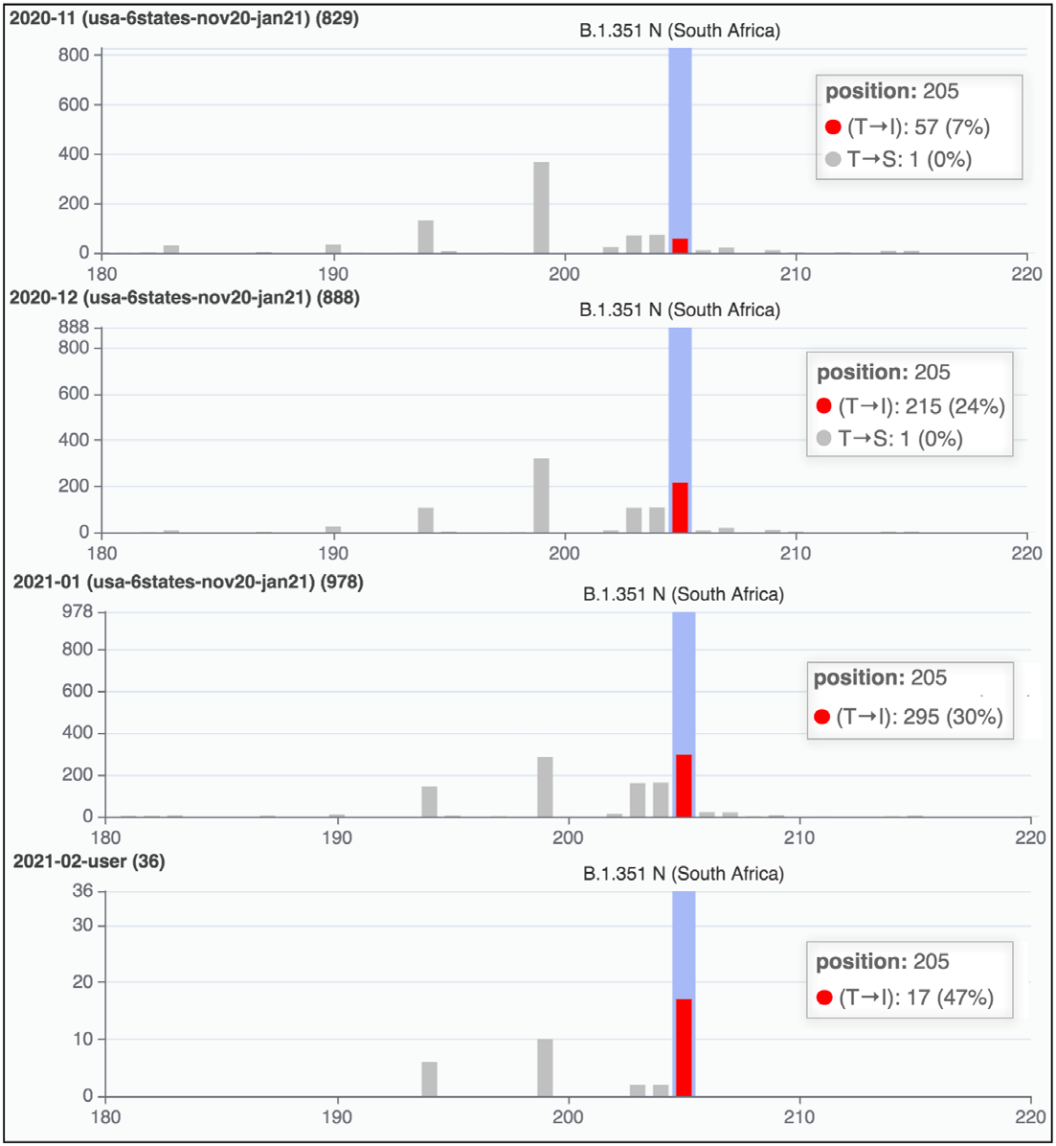
Comparison of three groups from the running example and an additional group derived from user-input batch of sequences, submitted to GenBank in February 2021. We observe the T205I change in the N protein; comparatively, bars show an increase of the percentage of the sequences carrying the variant. The figure has been built by zooming 1.5x the default Y-axis options and selecting the X-axis range 180–220 of the N protein.

We then compare these new recent sequences against the ones of the running example (submitted up to 29 January). To analyse the two projects together, we merge the current project with the previously saved running example project, whose three groups are displayed in Figure 6 marked as ‘usa-6states-nov20-jan21’; we also build a new group with all the sequences belonging to the user-input batch. In Figure 7, we comparatively inspect the four groups by focusing on a portion of the N protein, where we highlight one of the nine variants pertaining to the B.1.351 lineage (19) (named the *501.V2* or *South African variant*); even if the sample size of the last group is small, we observe that its presence is increasing over time. Three additional use cases can be found in the Supplementary Material (see Supplementary Figures S6–S12). They provide examples relative to SARS-CoV-2 variants of concern/interest (initially observed in UK, California and New York) based on ViruSurf. This material describes how new variants can be traced since their starting dates.

## DISCUSSION

The interest on the spreading of SARS-CoV-2 variants during the winter and spring seasons of 2021 is enormous, as variants are increasing the diffusion of the COVID-19 disease and may hamper the effectiveness of vaccination campaigns. The VirusViz system is a new powerful tool for supporting comparative visual analysis of variants, both of nucleotides and of amino acids, accepting input from both public sequences and user-provided sequences, integrated with a curated knowledge base of variants of interest; public sequences in ViruSurf and variants selected within the knowledge base are continuously updated. VirusViz is designed with a general approach, thus it can show variant distribution for other viral species. Although many other web tools can be used to inspect SARS-CoV-2 variants, VirusViz is the only tool currently supporting user-defined grouping of sequences for the comparative inspection of variant distributions across groups. We are in the process of developing a new data source, called EpiSurf, which integrates public epitopes from IEDB (20) with public amino acid sequences from ViruSurf. The new resource will be used to assess epitope’s conservancy (depending on the absence/presence of mutations), an important property in the design/assessment of vaccines, drugs, and serological assays. This further demonstrates the possibility of easily adding new data sources that use VirusViz for data visualization.

## DATA AVAILABILITY

The VirusViz web application is freely available at http://gmql.eu/virusviz/, without login requirements.

## Supplementary Material

gkab478_Supplemental_File
