# Supplementary material for: VirusViz: comparative analysis and effective visualization of viral nucleotide and amino acid variants
Source: Nucleic Acids Res. 2021 Jun 9;49(15):e90. doi: 10.1093/nar/gkab478 (PMC8344854; doi:10.1093/nar/gkab478)
Supplement: gkab478_Supplemental_File [file gkab478_supplemental_file.pdf]

VirusViz supplementary data

Anna Bernasconi [1,*], Andrea Gulino [1,*], Tommaso Alfonsi [1], Arif Canakoglu [1], Pietro Pinoli [1], Anna Sandionigi [2], and Stefano Ceri [1,†]

[1]Dipartimento di Elettronica, Informazione e Bioingegneria, Politecnico di Milano, Via Ponzio 34/5, 20133, Milano, Italy
[2]Quantia Consulting S.r.l., Via Petrarca 20, 22066, Mariano Comense, Como, Italy

[*]Co-first authors
[†]Corresponding author. Tel: +39 02 2399 3532; Fax: +39 02 2399 3411; Email: stefano.ceri@polimi.it

VirusViz JSON schema

ready
boolean default true
When false, the interface will periodically call again the service providing the json until the value of ready turns to true or an error is thrown (e.g. 404)

result
Result body

Result body

sequencesCount
integer
Number of sequences in the result.

taxon_id
integer
Taxonomy identifier of the virus (e.g. 2697049).

exclude_n
boolean default false
Exclude nucleotide variants.

exclude_a
boolean default false
Exclude amino acid variants.

schema
array Metadata attribute

Metadata attribute

name
string /^.*$/
attribute name

type
string /^.*$/
(optional) attribute type - possible values are 'categorical', 'numerical', 'lineage', 'date', 'location'. If not specified the type is automatically inferred.

sequences
Sequences
This part contains the information related to sequences. It is implemented as a dictionary of sequence IDs.

Sequences
This part contains the information related to sequences. It is implemented as a dictionary of sequence IDs.

[sequence_id]
Sequence

Sequence

id
string /^.*$/
Sequence identifier, as the dictionary key (i.e. = [sequence_id]).

sequenceFormat
string /^.*$/ default
Format used for encoding the sequence string specified in the 'sequence' property.

sequence
string /^.*$/ default
Plain text of the full sequence (sequenceFormat='plain') or base64 encoded GZIP compressed sequence ((sequenceFormat='gzip').

meta
Metadata
A dictionary of metadata, which keys appear in the schema.

Metadata
A dictionary of metadata, which keys appear in the schema.

[attribute_name]
string /^.*$/ default
value associated to the metadata attribute specified by [attribute_name] (e.g. if [attribute_name] = 'lineage' a possible value is 'B.1')

Figure S1: First part of the JSON file received in input by VirusViz

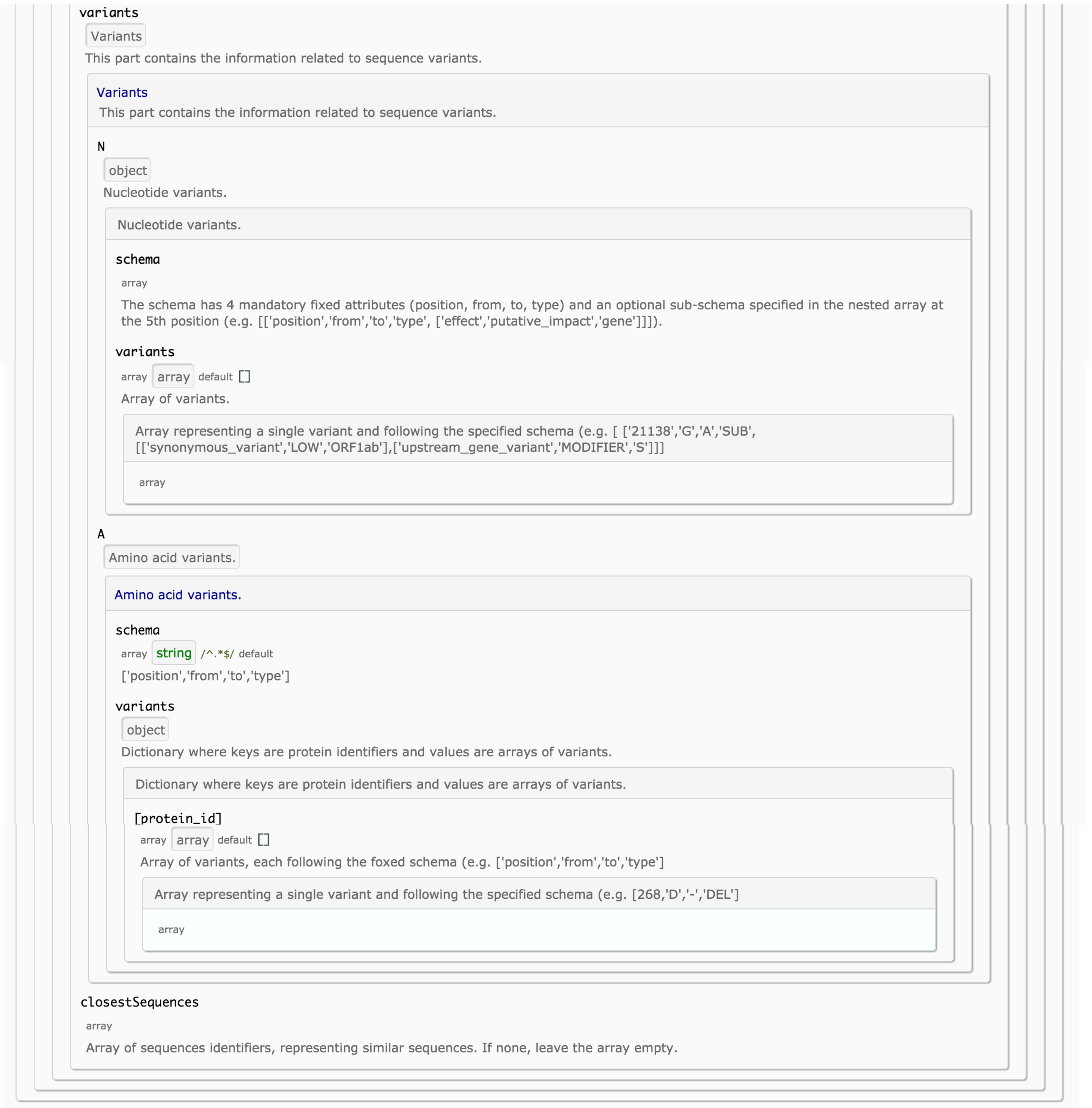

Figure S2: Second part of the JSON file received in input by VirusViz

```json
{
    "ready":true,
    "result":{
        "sequencesCount":1,
        "taxon_id":2697049,
        "exclude_n":false,
        "exclude_a":false,
        "schema":[
            {
                "name":"lineage",
                "type":"categorical"
            }
        ],
        "sequences":{
            "MW123456.1":{
                "id":"MW123456.1",
                "sequenceCompression":"plain",
                "sequence":"AAA..TTT",
                "closestSequences":[],
                "meta":{
                    "lineage":"L"
                },
                "variants":{
                    "N":{
                        "schema":[
                            "position",
                            "from",
                            "to",
                            "type",
                            [
                                "effect",
                                "putative_impact
                                "gene"
                            ]
                        ],
                        "variants":[
                            [
                                21138,
                                "G",
                                "A",
                                "SUB",
                                [
                                    [
                                        "synonymous_variant",
                                        "LOW",
                                        "ORF1ab"
                                    ],
                                    [
                                        "upstream_gene_variant",
                                        "MODIFIER",
                                        "S"
                                    ]
                                ]
                            ]
                        ]
                    },
                    "A":{
                        "schema":[
                            "position",
                            "from",
                            "to",
                            "type"
                        ],
                        "variants":{
                            "NSP2":[
                                [
                                    268,
                                    "D",
                                    "-",
                                    "DEL"
                                ]
                            ]
                        }
                    }
                }
            }
        }
    }
}
```


Figure S3: Example instance of the JSON session file in input by VirusViz, with one sequence.

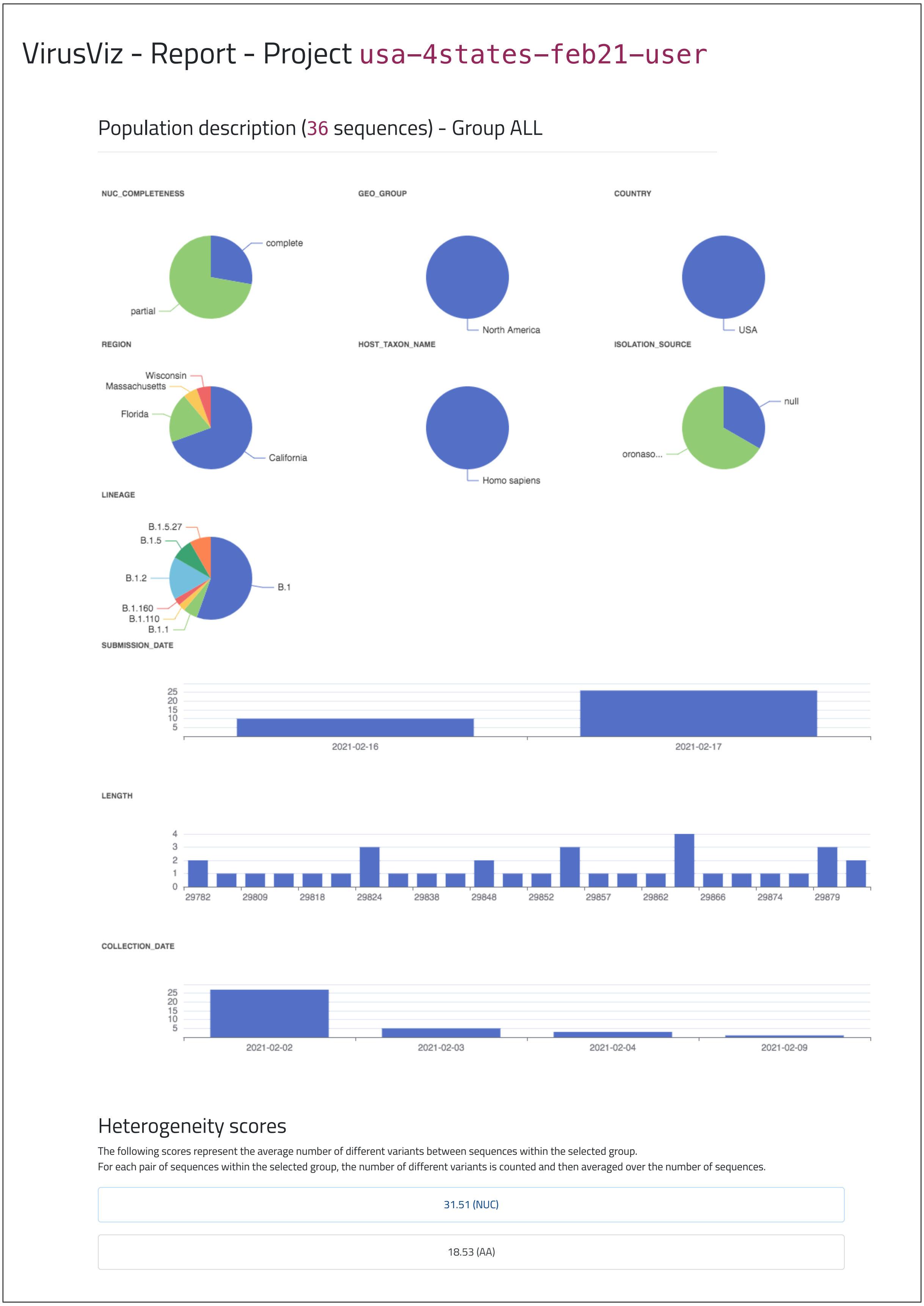

Figure S4: First page of *Population report* printed as a PDF document from the user's browser. It contains user notes and descriptive statistics on metadata, in the form of pie-charts and bar-plots.

Observed nucleotide variants

count	position	from	to	type
36	23403	A	G	SUB
36	14408	C	T	SUB
36	3037	C	T	SUB
36	241	C	T	SUB
28	25563	G	T	SUB

Observed amino acid variants

count	protein	position	from	to	type
36	Spike (surface glycoprotein)	614	D	G	SUB
36	NSP12 (RNA-dependent RNA polymerase)	323	P	L	SUB
28	NS3 (ORF3a protein)	57	Q	H	SUB
26	NSP2	85	T	I	SUB
17	N (nucleocapsid phosphoprotein)	205	T	I	SUB

Observed variants with associated effects

count	protein	position	from	to	type	effect : method	pub. DOI	other
36	Spike (surf...	614	D	G	SUB	higher fatality_rate	↗ ↗	-
						higher fatality_rate : Clin	-	↗
						higher infectivity	↗	-
						higher infectivity : Exp	↗	-
						higher infectivity : Inf	↗	-
						higher protein_stability	↗ ↗	↗
						higher protein_stability : Comp	↗	↗
						higher viral_transmission	↗ ↗ ↗	-
						higher viral_transmission : Inf	↗	-
						lower intraviral_protein_protein_interaction : Comp	-	↗
						lower protein_stability	↗	-
						unaffected disease_severity	↗	-
						unaffected fatality_rate	↗	-
						unaffected sensitivity_to_convalescent_sera	↗	-
36	NSP12 (R...	323	P	L	SUB	higher fatality_rate	↗	-
						higher fatality_rate : Clin	-	↗
						higher infectivity	-	↗
						higher protein_stability	↗	-
						higher protein_stability : Comp	↗	-
						higher viral_transmission	-	↗
						lower protein_flexibility : Comp	↗	-
						unaffected protein_stability : Comp	-	↗
28	NS3 (ORF...	57	Q	H	SUB	higher fatality_rate : Clin	-	↗
						higher infectivity	-	↗
						higher intraviral_protein_protein_interaction : Comp	↗	-
						higher protein_stability	-	↗
						higher viral_transmission	↗ ↗	↗
						higher viral_transmission : Inf	↗	-
						lower fatality_rate	↗	-
						lower protein_stability	↗	↗
						lower protein_stability : Comp	-	↗
26	NSP2	85	T	I	SUB	lower protein_stability	↗	-
16	Spike (surf...	452	L	R	SUB	higher binding_affinity_to_host_receptor : Comp	↗	-
						lower sensitivity_to_neutralizing_mAbs : Exp	↗	-

Figure S5: Second page of *Population report* printed as a PDF document from the user's browser. It contains a measure of the heterogeneity of the population and tables of present nucleotide and amino acid variants (with known effects).

The birth of the UK variant

According to a tweet by Emma Hodcroft [1] posted on December 14th, 2020, a new variant was observed in the United Kingdom, showing a specific combination of co-occurring amino acid changes (namely a substitution N501Y and two deletions at positions 69 and 70 of the Spike protein). Shortly after, the variant was better defined as a wider set of co-occurring amino acid changes in a post on Virological (a virology forum widely adopted by the scientific community) [2] and later declared a Variant of Concern with code VOC-202012/01 [3]. To reproduce the growth of this variant, on May 2nd, 2021 we retrieved from ViruSurf 64850 good quality sequences (we set a condition forcing the number of unknown nucleotides to be $< 5\%$), collected in United Kingdom from mid-October until mid-December.

On VirusViz, we then prepared four groups, each containing sequences collected in a two-week period (second half of October, first and second half of November, first half of December), and opened the four groups on the *Group comparison* page on the full Spike protein, so to observe the behavior of the N501Y amino acid change. As shown in Figure S6, we noted a clear increase, from the second half of October (1% of available sequences present the mutation), to the first half of November (5% of available sequences present the mutation), throughout the second half (13%), and considerably growing during the first half of December (36%). Note that we highlighted with blue vertical lines the amino acid changes belonging to the B.1.1.7 lineage, available as a predefined region in the *Region definition* page. They correspond to amino-acid changes of the UK variant, which have now become well known (as of May 2021, the UK variant is dominant in UK and many other countries).

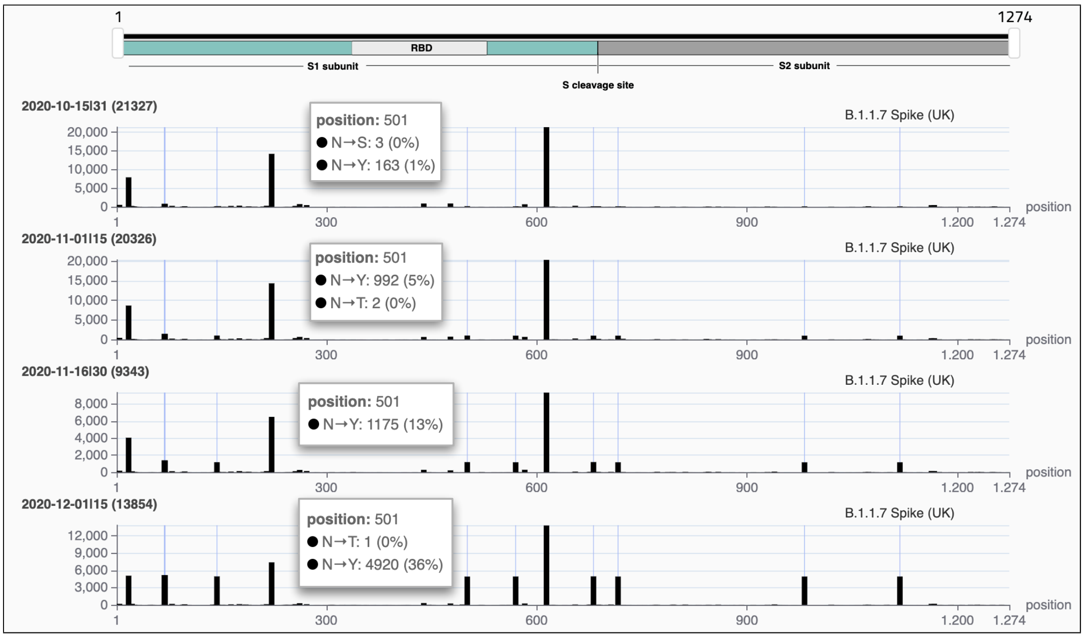

Figure S6: Comparison among Spike protein amino acid variants in four different periods in the UK.

Then, on VirusViz, we also observed the overall distribution during this two-months period in England, Wales and Scotland, reported as three different regions of the UK country. We built three groups by a metadata selection on the ‘region’ metadata attribute; the comparison shown in Figure S7 indicates that the change was most present in England (44%) than in Scotland (10%) and Wales (6%).

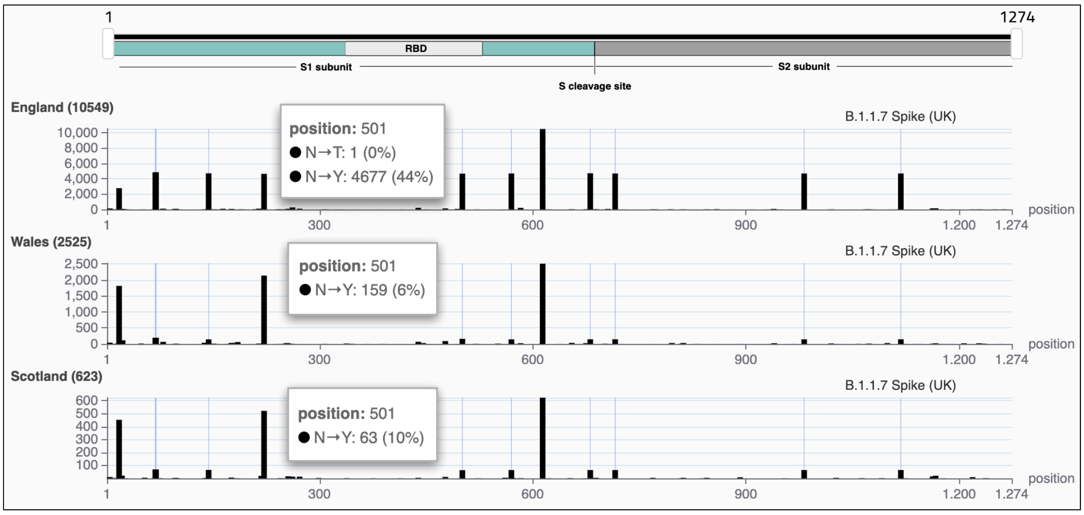

Figure S7: Comparison among Spike protein amino acid variants in England, Wales and Scotland during the overall period mid-October through mid-December.

The growth of the Californian variant

From a JAMA Network research letter [4] dated February 11, 2021, we read that a novel Californian variant is increasing its presence. This is defined by five mutations:

- ORF1a: I4205V (corresponding to I65V in NSP9),
- ORF1b: D1183Y (corresponding to D206Y in NSP13),
- Spike: S13I,
- Spike: W152C,
- Spike L452R

and has been designated as CAL.20C, or 20C/S:452R (according to NextStrain [5] nomenclature), or B.1.429 (using Pangolin lineages [6]). To reproduce the growth of this variant, on May 2nd, 2021 we retrieved from ViruSurf 25436 sequences, collected in California from November 2020 until February 2021. We then built four separate groups, one for each month in the given period, and compared their variant distributions. The region highlighted in blue, one of the predefined regions in the *Region definition* page, indicates amino acid changes that represent the variant for the three proteins Spike, NSP9, and NSP13. In Figure S8, we can appreciate the result from VirusViz for the Spike protein (positions 13, 152, and 452). In Figure S9, see the results for NSP9 (position 65). In Figure S10, see the results for NSP13 (position 206).

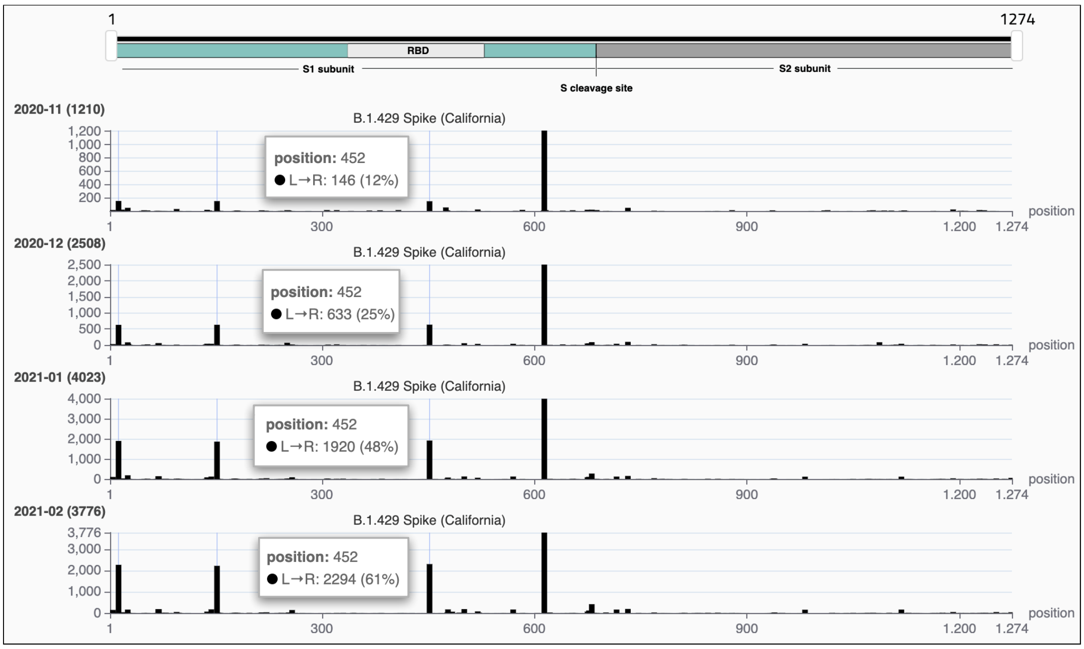

Figure S8: Comparison among Spike protein amino acid variants in four different periods in California.

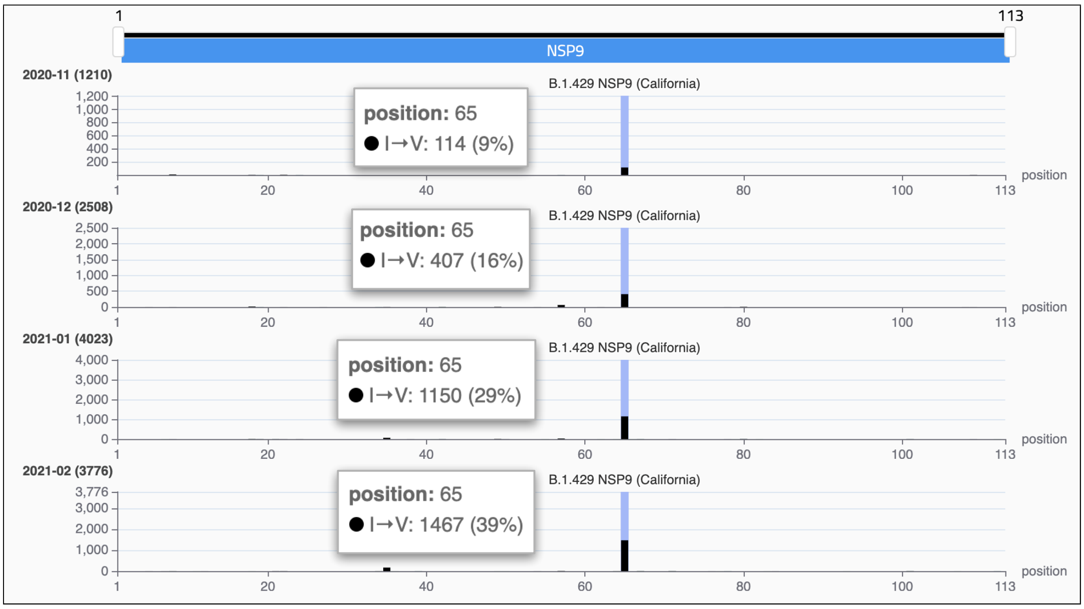

Figure S9: Comparison among NSP9 nonstructural protein amino acid variants in four different periods in California.

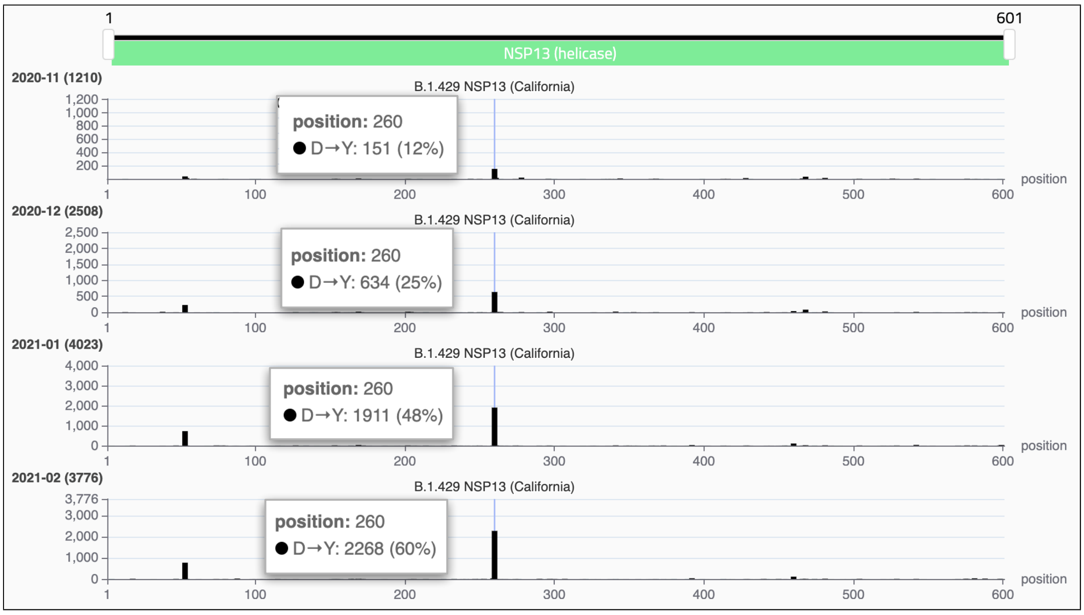

Figure S10: Comparison among NSP13 nonstructural protein amino acid variants in four different periods in California.

New York comparisons

From the CDC Variant Report [7] updated on May 5th, 2021 we learn that a novel variant, namely B.1.526, has become “of interest”, as it is increasing its presence. This has been informally called “New York variant”, as it was first detected in this US state. With VirusViz we confirmed this increased presence: on May 2nd, 2021 we downloaded 3918 sequences from ViruSurf collected in New York State. First, we used the *Group comparison* functionality by grouping all the retrieved sequences according to their ‘lineage’ metadata attribute. In Figure S11, we show those lineages with the highest number of occurrences in the full population of sequences; note that lineages B.1.1.7 (UK, marked with a green rectangle) and B.1.429/B.1.427 (California, marked with a blue rectangle) are present, but also note lineages B.1.526, B.1.526.1 and B.1.526.2 (red rectangle), which are collectively the most numerous. Then, in Figure S12 we focus on the distributions of these three lineages, that are currently reported as Variants of Concern or of Interest in the CDC report: B.1.1.7, B.1.429, and B.1.526.

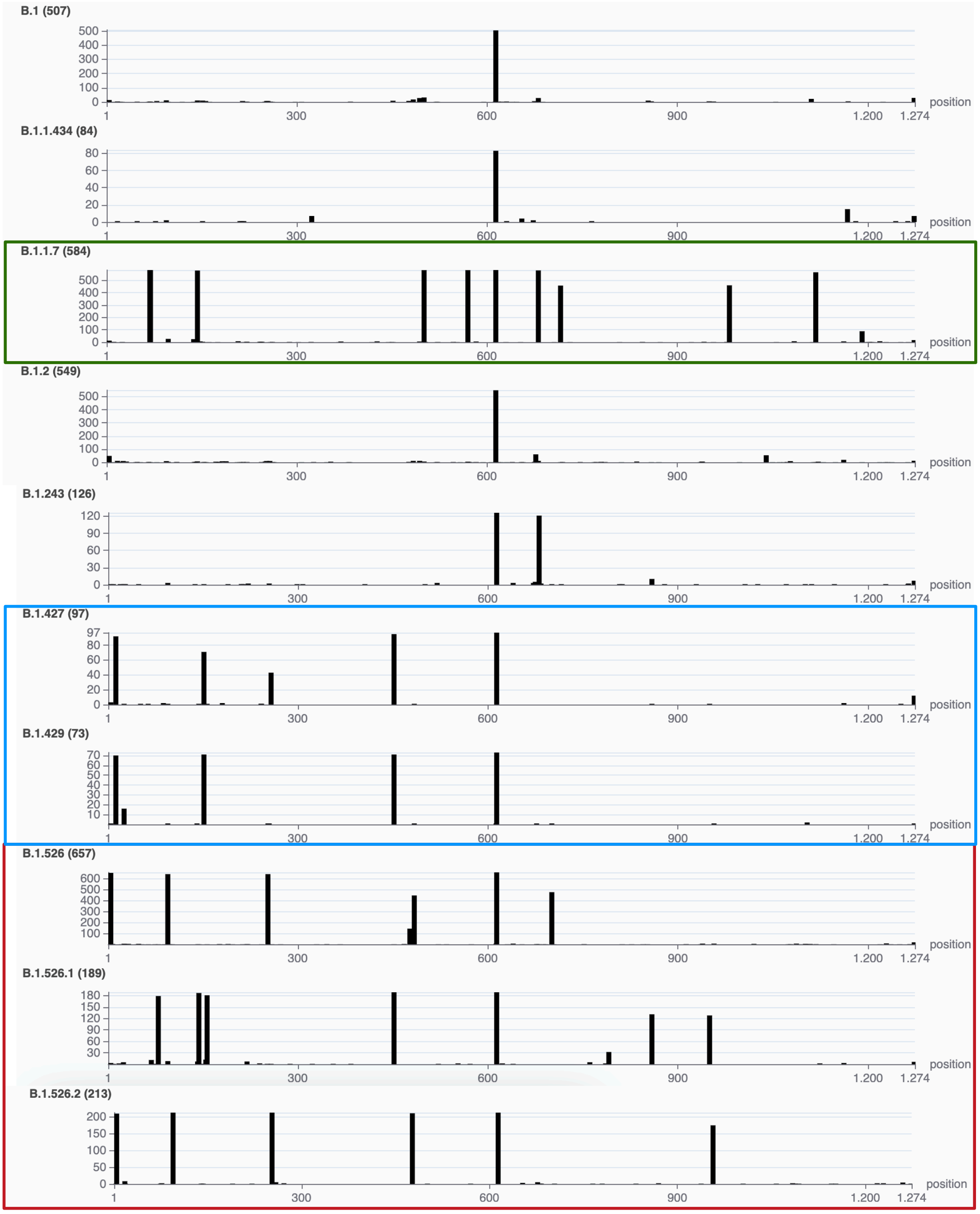

Figure S11: Comparison on the Spike protein of the amino acid variants in the ten most highly present lineages in New York state.

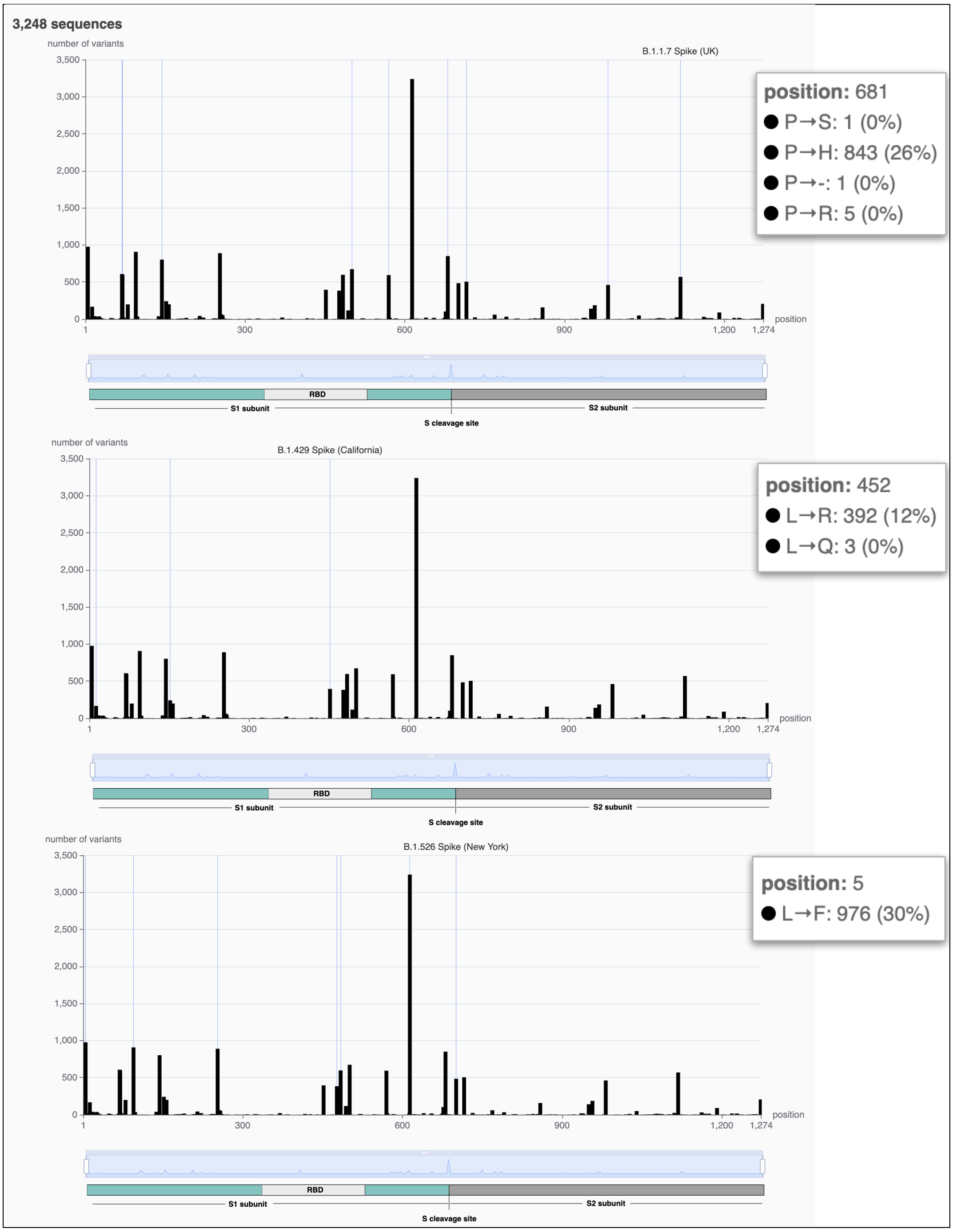

Figure S12: Distribution of Spike protein amino acid changes. In the first panel we highlight the region corresponding to characteristic mutations of B.1.1.7 lineage, in the second panel of B.1.429, in the third panel of B.1.526. For each panel, we show – in the card on the right – the percentages of the most present changes in the lineage.

References

[1] Hodcroft, E. Tweet of December 14th, 2021. https://twitter.com/firefoxx66/status/1338533710178775047 Accessed online on May 9th, 2021.

[2] Rambaut, A., Loman, N., Pybus, O., Barclay, W., Barrett, J., Carabelli, A., Connor, T., Peacock, T., Robertson, D. L., and Volz, E. on behalf of COVID-19 Genomics Consortium UK (CoG-UK). Preliminary genomic characterisation of an emergent SARS-CoV-2 lineage in the UK defined by a novel set of spike mutations. https://virological.org/t/preliminary-genomic-characterisation-of-an-emergent-sars-cov-2-lineage-in-the-uk-defined-by-a-novel-set-of-spike-mutations/563 Accessed online on May 9th, 2021.

[3] COVID-19 Genomics Consortium UK (CoG-UK) COG-UK report on SARS-CoV-2 Spike mutations of interest in the UK, January 15th, 2021. https://www.cogconsortium.uk/wp-content/uploads/2021/01/Report-2_COG-UK_SARS-CoV-2-Mutations.pdf Accessed online on May 9th, 2021.

[4] Zhang, W., Davis, B. D., Chen, S. S., Sincuir Martinez, J. M., Plummer, J. T., and Vail, E. (Apr, 2021) Emergence of a Novel SARS-CoV-2 Variant in Southern California. *JAMA,* **325**(13), 1324–1326 [PubMed Central:PMC7879386] [DOI:10.1001/jama.2021.1612] [PubMed:33571356].

[5] Hadfield, J., Megill, C., Bell, S. M., Huddleston, J., Potter, B., Callender, C., Sagulenko, P., Bedford, T., and Neher, R. A. (12, 2018) Nextstrain: real-time tracking of pathogen evolution. *Bioinformatics,* **34**(23), 4121–4123 [PubMed Central:PMC6247931] [DOI:10.1093/bioinformatics/bty407] [PubMed:29340210].

[6] Rambaut, A., Holmes, E. C., O'Toole, A., Hill, V., McCrone, J. T., Ruis, C., du Plessis, L., and Pybus, O. G. (11, 2020) A dynamic nomenclature proposal for SARS-CoV-2 lineages to assist genomic epidemiology. *Nat Microbiol,* **5**(11), 1403–1407 [DOI:10.1093/molbev/mst024] [PubMed:32669681].

[7] Centers for Disease Control and Prevention (CDC) SARS-CoV-2 Variant Classifications and Definitions. https://www.cdc.gov/coronavirus/2019-ncov/cases-updates/variant-surveillance/variant-info.html Accessed online on May 9th, 2021.
